# Supplementary material for: Gut microbiome affects the metabolism of metronidazole in mice through regulation of hepatic cytochromes P450 expression
Source: PLoS One. 2021 Nov 9;16(11):e0259643. doi: 10.1371/journal.pone.0259643 (PMC8577747; doi:10.1371/journal.pone.0259643)
Supplement: S1 Data — (DOCX) [file pone.0259643.s001.docx]

**S1 Data.**

The individual plasma concentrations of metronidazole and 2-hydroxymetronidazole

| ***Metronidazole*** | concentration (µM) |
| --- | --- |
| 1 GF mouse 0 h | 0 |
| 2 GF mouse 0 h | 0 |
| 3 GF mouse 0 h | 0 |
| 4 GF mouse 0 h | 0 |
| 5 GF mouse 2 h | 108.24 |
| 6 GF mouse 2 h | 144.27 |
| 7 GF mouse 2 h | 134.31 |
| 8 GF mouse 2 h | 205.69 |
| 9 GF mouse 6 h | 44.53 |
| 10 GF mouse 6 h | 31.00 |
| 11 GF mouse 6 h | 37.17 |
| 12 GF mouse 6 h | 59.75 |
| 13 GF mouse 24 h | 0 |
| 14 GF mouse 24 h | 0 |
| 15 GF mouse 24 h | 3.14 |
| 16 GF mouse 24 h | 0 |

| ***Metronidazole*** | concentration (µM) |
| --- | --- |
| 1 SPF mouse 0 h | 0 |
| 2 SPF mouse 0 h | 0 |
| 3 SPF mouse 0 h | 0 |
| 4 SPF mouse 0 h | 0 |
| 5 SPF mouse 2 h | 101.20 |
| 6 SPF mouse 2 h | 133.46 |
| 7 SPF mouse 2 h | 49.37 |
| 8 SPF mouse 2 h | 112.27 |
| 9 SPF mouse 6 h | 45.91 |
| 10 SPF mouse 6 h | 27.20 |
| 11 SPF mouse 6 h | 28.53 |
| 12 SPF mouse 6 h | 30.73 |
| 13 SPF mouse 24 h | 0.20 |
| 14 SPF mouse 24 h | 0 |
| 15 SPF mouse 24 h | 0.15 |
| 16 SPF mouse 24 h | 0 |

| ***2-hydroxymetronidazole*** | concentration (µM) |
| --- | --- |
| 1 GF mouse 0 h | 0 |
| 2 GF mouse 0 h | 0 |
| 3 GF mouse 0 h | 0 |
| 4 GF mouse 0 h | 0 |
| 5 GF mouse 2 h | 12.46 |
| 6 GF mouse 2 h | 13.84 |
| 7 GF mouse 2 h | 11.45 |
| 8 GF mouse 2 h | 19.89 |
| 9 GF mouse 6 h | 12.84 |
| 10 GF mouse 6 h | 7.77 |
| 11 GF mouse 6 h | 9.12 |
| 12 GF mouse 6 h | 13.45 |
| 13 GF mouse 24 h | 0 |
| 14 GF mouse 24 h | 0 |
| 15 GF mouse 24 h | 0 |
| 16 GF mouse 24 h | 0 |

| ***2-hydroxymetronidazole*** | concentration (µM) |
| --- | --- |
| 1 SPF mouse 0 h | 0 |
| 2 SPF mouse 0 h | 0 |
| 3 SPF mouse 0 h | 0 |
| 4 SPF mouse 0 h | 0 |
| 5 SPF mouse 2 h | 12.57 |
| 6 SPF mouse 2 h | 13.26 |
| 7 SPF mouse 2 h | 21.87 |
| 8 SPF mouse 2 h | - |
| 9 SPF mouse 6 h | 8.96 |
| 10 SPF mouse 6 h | 11.36 |
| 11 SPF mouse 6 h | 12.23 |
| 12 SPF mouse 6 h | 10.62 |
| 13 SPF mouse 24 h | 0 |
| 14 SPF mouse 24 h | 0 |
| 15 SPF mouse 24 h | 0 |
| 16 SPF mouse 24 h | 0 |
