# Supplementary material for: Gut microbiome affects the metabolism of metronidazole in mice through regulation of hepatic cytochromes P450 expression
Source: PLoS One. 2021 Nov 9;16(11):e0259643. doi: 10.1371/journal.pone.0259643 (PMC8577747; doi:10.1371/journal.pone.0259643)
Supplement: Attachment — Submitted filename: Reviewer response.docx [file pone.0259643.s003.docx]

Manuscript ID: PONE-D-21-19140

Title: Gut microbiome affects the metabolism of metronidazole in mice through regulation of hepatic cytochromes P450 expression

The manuscript described the effect of gut microbiome on the fate of metronidazole by profiling the metronidazole’s pharmacokinetics changes (Cmax and metabolic ratio). And the selected P450 enzymes related changes (mRNA expression, protein expression and enzyme activity) were compared in both SPF and germ-free (GF) mice after administration metronidazole within 24 hours. And the results provided the possibility of the selected hepatic P450 enzymes altered metronidazole metabolism.

1. There were some wording issues e.g. CYP (line171) and one cited literature [36] information about metronidazole has not been found within the mentioned literature content.

2. The structure and enzymatic information that mentioned in the introduction section was not clearly marked in the Figure 1.

3. Some antibiotics mechanism that mentioned in the manuscript (Introduction section) has been summarized in the related review [1] and should be mentioned in the introduction.

4. A lot of details of Section ‘cytochrome P450 enzyme activity assays’ were missing. Such as the particle size of column; the UV/ fluorescence detection parameters, and the gradient ranges.

5. The metabolic ratios were mentioned with only the values (line 234-238). Did the metabolic ratio calculate individually? What is the mean and SD for the metabolic ratio?

6. Could the Figure 3-5 changed into a box plot with individual values plus mean and SD to facilitate the data trend observation?

7. Literature has reported that co-dosing metronidazole with neomycin, vancomycin, and ampicillin for 3 weeks could obtain pseudo germ-free mice [2]. Could the short-term treatment with metronidazole in SPF mice regulate similar metabolic pathway? If so, did the comparison between SPF mice and GF mice after administration metronidazole describe the difference between pseudo germ-free mice versus germ-free mice metabolic pathway?

8. The pharmacokinetic data showed in Figure 2 have a clear overlap in Cmax value. Especially, the SD of the GF mice Cmax approached the SPF mice Cmax mean value. Was there a possibility that there is an outlier plasma concentration within the limited sample numbers? Could the individual Cmax values be attached as supplementary data?

9. Besides the described results, were there any RNA-seq screening data and/or proteomics data results support the selected metabolic enzyme regulation? Or does there any other metabolic enzyme also significantly involved into the metronidazole metabolism?

Reference

[1] Leitsch D. A review on metronidazole: an old warhorse in antimicrobial chemotherapy. Parasitology. 2019 Aug;146(9):1167-78.

[2] Liang W, Zhao L, Zhang J, Fang X, Zhong Q, Liao Z, Wang J, Guo Y, Liang H, Wang L. Colonization Potential to Reconstitute a Microbe Community in Pseudo Germ-Free Mice After Fecal Microbe Transplant From Equol Producer. Frontiers in Microbiology. 2020 Jun 5;11:1221.
