# Supplementary material for: Gut microbiome affects the metabolism of metronidazole in mice through regulation of hepatic cytochromes P450 expression
Source: PLoS One. 2021 Nov 9;16(11):e0259643. doi: 10.1371/journal.pone.0259643 (PMC8577747; doi:10.1371/journal.pone.0259643)
Supplement: Attachment — Submitted filename: Response to reviewers.docx [file pone.0259643.s005.docx]

Dear Editor,

thank You for your kind revision and suggestions for improvement of our paper. We wish to thank reviewers as well for their helpful comments and for taking the time to point out options to improve our manuscript.

Below, we have addressed the comments of Reviewer #3:

**1. Better and individual animal western blot bands are requested.**

Unfortunately, we are not able to show Western blots for individual animals. The reason is that the experiment has been realized with SPF and germ-free (GF) mice to document the differences in response caused of presence of the microbiome. In fact, the GF animal model is very unique (and only few laboratories in the world have access to this model) and we were limited by number of animals and by the amount of experimental material (small murine livers). On the other hand, there was the need to get the information on the activities of individual CYP forms. This is why we pooled the samples to get as much information as possible (enzyme activities, protein and mRNA expression). We deal with this issue in every experiment with murine livers obtained with the germ-free models. We feel that even when the microsomes are pooled, the results and corresponding figures support the conclusions reached.

**2. Figures should be arranged in order with results.**

We thank very much the Reviewer for bringing this issue to our attention. We moved the legends of the figures after the corresponding description in the results.

**3. Some results and Figures (order) are not match.**

Thank you for the comment. We apologize for the mistake and we corrected the number of the figure in the manuscript.

**4. Missing X-axis in Figure 4 C and G.**

The reviewer is right that the figure C and G differ from the other graphs. However, description of X-axis is not missing here as it is only in a different position due to SD reaching the minus values.

We would like to say again that we appreciate all your time given to our article.

With best regards

Lenka Jourová, Ph.D.

Department of Medical Chemistry and Biochemistry

Faculty of Medicine and Dentistry, Palacký University

Hněvotínská 3

775 15 Olomouc, Czech Republic

Tel: +420 585 632 346, email: LenkaJourova@seznam.cz
