# Supplementary material for: Gut microbiome affects the metabolism of metronidazole in mice through regulation of hepatic cytochromes P450 expression
Source: PLoS One. 2021 Nov 9;16(11):e0259643. doi: 10.1371/journal.pone.0259643 (PMC8577747; doi:10.1371/journal.pone.0259643)
Supplement: Attachment — Submitted filename: Reviewer comments plos one P450 metronidazole.docx [file pone.0259643.s002.docx]

This manuscript has used germ free (GF) and specific pathogen free (SPF) to demonstrate that the absence of gut microbiota appeared to result in a temporal trend of increase (although not statistically significant) in the plasma metronidazole 2 hours after oral administration, but not at other time points. The hydroxylated metabolite determined was not changed. In addition, this study demonstrated that oral metronidazole the mRNA expression of selected P450s (CYP1-3) and xenobiotic-sensing transcription factors to a different extent between SPF and GF mice, although common patterns were also observed such as an up-regulation of Cyp2b10 by metronidazole in both mouse groups, over the time course. In contrast, the protein and enzyme activities were not readily altered significantly, although certain trend of increase or decrease was observed.

Major points:

1. Figure 2 – Figure 5: it is strongly recommended that the SPF and GF data should be plotted in the same figure, so one can evaluate the basal differences between SPF and GF. If this results in too many bars in one figure, the plots can be split per time point. This is important because it is highly possible that the tendency in the change in the metronidazole pharmacokinetics is not due to the differential response patterns of the P450s to metronidazole treatment between SPF and GF, but simply due to the basal differences in these enzymes between SPF and GF.

2. Please show the western blot result as the actual immuno-blots (currently Table 1), so that one can evaluate the specificity of the bands, sample size, and biological variations. Please include the standard deviations or standard errors in Table 1.

3. Method section: The RT-qPCR normalization method claimed that the data were expressed as ddCq and normalized to the Hprt house-keeping gene. However, It looks like Figure 3 and Figure 4 actually artificially assigned both SPF control and GF control to 1. This might not be the best way to express the data because the SPF control may be different from the GF control. Please consider re-plotting the data so it matches the Method section.

4. Method section: statistical method: one-way ANOVA was performed. However, this study design has two factors, namely enterotype (SPF vs. GF) and treatment (control vs. metronidazole over a time course). Thus two-way ANOVA is a more appropriate method.

5. From the plasma metronidazole result (Figure 2A), I think the 2h time point is the most critical for evaluation regarding mRNA expression, protein levels, and enzyme activities. Please especially take a look into this time point to connect the Figure 2A result with the other figures.

6. Figure 2A and 2B: because the hydroxylated metabolite did not decrease to a similar extent as the apparent increase of the parent compound, it is possible that this may be a disposition issue. Please consider measuring hepatic metronidazole and its metabolite (and maybe even urine). Also one needs to consider transporters and Ugts in addition to the P450s.

7. Introduction section says CYP2A6 in humans is the most predominant enzyme for metronidazole metabolism. However the mouse data on the Cyp2a isoforms do not seem to explain the trend of increase in plasma metronidazole at 2 hours; in fact there was a clear trend of decrease in CYP2A enzyme activities to a greater extent in SPF mice than in GF mice (opposite to the expectations) at 2h. The mRNA increase in Cyp2a was only observed after 6h and is not likely responsible for the increase in plasma metronidazole at 2h. Would other P450 isoforms be responsible for the mouse metabolism of this drug? Please discuss based on your findings.

Minor points:

1. Method section: Western blot: please include the catalog numbers of the antibodies.

2. Method section: please define the age, sex, and sample size of the mice.

3. Abstract: please be more specific in describing the key findings on mRNA, protein, and enzyme activities.

4. Title: it seems that the study was mainly determining the effect of the absence of gut microbiome on the hepatic P450s, not necessarily the effect of the gut microbiome on the hepatic P450s.

5. Typo: “could not be determinate.” 🡪 “ could not be determined.”
